# Parental perceptions and beliefs about childhood asthma: a cross-sectional study

**DOI:** 10.3325/cmj.2011.52.637

**Published:** 2011-10

**Authors:** Rola Zaraket, Mohamad A Al-Tannir, Aref A Bin Abdulhak, Ahmad Shatila, Hani Lababidi

**Affiliations:** 1 Department of Pediatrics, Makassed General Hospital, Beirut, Lebanon; 2 Scientific Research and Publication Centre, King Fahd Medical City, Riyadh, Saudi Arabia; 3 Internal Medicine Department, King Fahd Medical City, Riyadh, Saudi Arabia; 4 Pulmonary and Critical Care Department, King Fahd Medical City, Riyadh, Saudi Arabia

## Abstract

**Aim:**

To assess parental perceptions and beliefs about asthma in children.

**Methods:**

We invited 6000 children aged 3 to 15 years from different schools in Lebanon to participate in the study from September 2007 to May 2008. In the first phase, in order to determine the prevalence of asthma in children, parents of all participating children filled out a small questionnaire. In the second phase, only parents of children with asthma filled out a detailed questionnaire about their perceptions of asthma.

**Results:**

Phase I included parents of 4051 children, 574 (14%) of whom had asthma and were recruited to phase II. Out of these, 389 parents entered the final data analysis. Around 54% of parents believed that asthma was hereditary and 7% believed it was contagious. When asked about triggering factors, 51% stated virus infection, 75% dust, and 17% food. Sixty percent of children with asthma lived with someone who smoked. Sixty-seven percent of parents believed that herbs had a role in asthma treatment and only 49% received asthma education. There was a significant difference in education level (*P* = 0.01) between the parents who denied the label of asthma (79%) and those who accepted it (21%). Sixty-seven percent of parents preferred oral over inhaler treatment, 48% believed inhalers were addictive, 56% worried about inhalers’ side effects, and 76% worried about using inhaled corticosteroids. Significantly more parents from rural (53%) than from urban areas (38%) believed that inhalers were addictive (*P* = 0.004).

**Conclusion:**

Parents of children with asthma had considerable misperceptions about the use of inhalers and the safety of inhaled corticosteroids. To improve asthma care in children, it is necessary to provide adequate education to parents.

Asthma is a common chronic disease and a major public health problem, especially among pediatric population (1-5). Bronchial asthma is defined as a chronic inflammatory disorder of the airways. The chronic inflammation causes an associated increase in airway hyper-responsiveness that leads to recurrent episodes of wheezing, breathlessness, chest tightness, and coughing, particularly at night or in the early morning. These episodes are usually associated with widespread but variable airflow obstruction that is often reversible either spontaneously or with treatment (6). In Lebanon and other Middle East countries, the terms chest allergy or recurrent dyspnea are often popularly used instead of asthma, probably to avoid the social stigma associated with chronic nature and functional impairment of the disease. Asthma has a hereditary basis but environment factors have also been implicated in its pathogenesis (7). Traditionally, it has been treated by allergen avoidance, beta-2 agonists, and inhaled corticosteroids. Despite improved understanding of the pathophysiology of asthma and the development of new therapeutic strategies, its incidence and related mortality and morbidity are still increasing annually in developed countries (1,8-13). It has been found that compliance with the National Heart, Lung, and Blood Institute guidelines is influenced by patient income and level of education (14) and that asthma incidence is higher among children from low socioeconomic class (8). Also, uncontrolled asthma was associated with low maternal education and parental concerns about adverse effects of medication (15). Optimal management can be achieved by educating patients and their families to make life-style changes and adhere to drug therapy for a long period even when symptoms are not present (15,16). This study was conducted to investigate beliefs, knowledge, and perceptions of parents in Lebanon toward asthma and its management.

## Methods

After having received approval from the Institutional Review Board at Makassed General Hospital, from September 2007 to May, 2008 we enrolled children aged 3 to 15 years from 10 schools located in 5 districts of Lebanon; two schools from each district with different socioeconomic status and demographic characteristics.

This cross-sectional study consisted of two phases; in the first phase, the parents of 6000 children were distributed a short questionnaire in order to determine whether their children suffered from asthma, chest allergy, or recurrent dyspnea. Those who checked one of these choices were recruited to the second phase. In the second phase, we used a more detailed, self administered questionnaire consisting mainly of closed-ended questions (yes/no). We collected data on child’s age and sex, and parental age and employment information. We also assessed parents’ perception of etiology of asthma (hereditary or contagious), triggers (dust, viral infection, or food), predisposing factors (indoor smoking and/or pets), and different modalities of asthma treatment (herbs, oral, or inhaled).

The use of medications was determined by asking parents to list prescription asthma medications they had at home and their frequency of use. Also, parents were asked about their worries and concerns about the use of inhaled corticosteroids. The adequacy of asthma treatment was evaluated on the basis of whether the child visited the physician regularly, the number of school days of absence, frequency of hospitalization, and awareness of symptoms of uncontrolled asthma including nocturnal dyspnea, repetitive cough, exercise dyspnea, and dyspnea at rest. Parents were also asked if they had received any form of education or explanation about asthma and its management from their physicians (web-extra material))(web extra material 1).

### Statistical analysis

Data are presented as number (%), mean ± standard deviation, and median (range). We used χ^2^ test to calculate the two tailed *P*-value and to determine the difference between parents who denied and accepted the label of asthma, parents from rural and urban areas, and relation between these characteristics and variables such as etiology (hereditary and contagious), triggers (viral, dust, and food), indoor smoking, treatment with herbs, parental attitudes on treatment (preference of syrup over inhaler, worries about inhalers addiction, side effects, and inhaled steroids), and asthma education. *P* values lower than 0.05 were considered significant. Statistical analysis was performed using SPSS, version 15.0 (SPSS Inc., Chicago, IL, USA).

## Results

**Figure 1:**
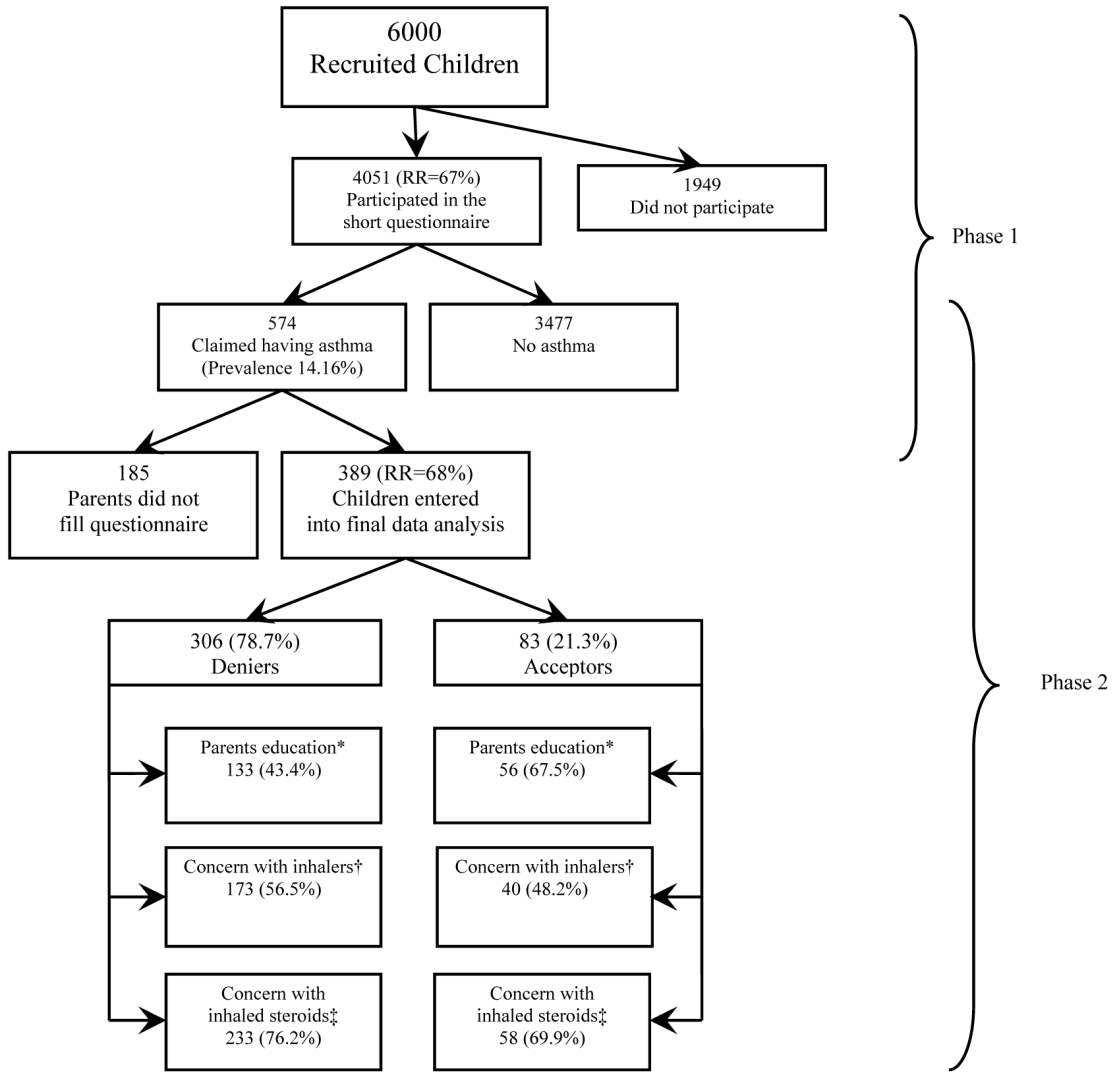
Two phases of the survey. χ^2^ test was used for comparisons. Asterisk represents *P* = 0.001; dagger represents *P* = 0.22; double dagger represents *P* = 0.30.

We gave a short questionnaire to 6000 parents of school children aged 3 to 15 years from different Lebanese districts. Out of 4051 (67%) children whose parents responded, 574 had asthma (asthma prevalence of 14%). Only 389 parents completed the second phase questionnaire and entered the final data analysis (response rate 68%) (Figure 1).

The characteristics of 389 children with asthma are presented in Table 1. Their mean age was 8.37 ± 2.68 years, and the male/female ratio was 1.26. As the most common symptom of uncontrolled asthma in children, 222 (76%) parents reported repetitive cough. Hundred and one (30%) parents recalled that their children had been hospitalized for asthma, more than a half (54.6%) of whom reported multiple admissions.

**Table 1:** Characteristics of Lebanese children with asthma enrolled in this study and their parents

Characteristics	No. (%) of participants
Age in years (mean ± standard deviation):	
child	8.37 ± 2.68
father	42.0 ± 7.3
mother	35.3 ± 6.5
Sex of children:	
female	172 (44)
male	218 (55.9)
Social class:*	
class I	67 (17.2)
class II	120 (30.8)
class III	192 (49.2)
Settlement size:	
urban	223 (57.2)
rural	167 (42.8)
Symptoms of uncontrolled asthma:	
repetitive cough (daily)	222 (76)
nocturnal dyspnea (more than one night/week)	152 (64.4)
exercise dyspnea	139 (61.2)
dyspnea at rest	49 (25.9)
Hospitalization for asthma (n = 338)	101 (29.9)
Absence from school	228 (68.3)
Regular visit to a physician (every one-three months)	125 (37.4)
Consulting pediatrician about the attack	225 (69.0)
Ability to treat the attack at home	229 (70.7)

*Class I – highly skilled workers (ie, physician, manager, teacher, pharmacist, shopkeeper); Class II – skilled workers (ie, clerk, factory foreman); Class III – semi-skilled and non-skilled workers (ie, taxi driver, factory worker, salesman, porter, laborer, waiter).

Only 82 (21%) parents identified asthma by name, the majority 249 (64%) referred to it as chest allergy, and 58 (15%) as dyspnea. The majority of them believed that asthma was a hereditary disease and cited exposure to dust as the most important trigger. About 174 (48%) parents worried that inhaler therapy may cause addiction and 216 (56%) worried about the side effects of inhalers (Table 2). Only 52 (24%) children were using inhaled corticosteroids and 90 (40%) antihistamines (Table 3). Important factors associated with concerns about inhaler therapy were low socioeconomic class, rural residency, and lack of asthma education (Table 4). Three hundred and six (78.7%) parents refused to label their children as having asthma (Figure 1).

**Table 2:** Perceptions about asthma of parents of Lebanese children with asthma according to settlement size

Parental perception of	Total number (%) of participants(n = 389)	No. (%) of participants living in		P*
		urban areas (n = 222)	rural areas (n = 167)	
**Etiology:**				
**hereditary**	211 (54.2)	139 (62.6)	72 (43.1)	0.001
**contagious**	28 (7.2)	6 (2.7)	22 (13.2)	0.001
**Triggers:**				
**viral infection**	200 (51.4)	120 (54.1)	80 (47.9)	0.26
**dust**	292 (75.0)	169 (76.1)	123 (73.7)	0.64
**food**	67 (17.2)	44 (19.8)	23 (13.8)	0.14
**indoor smoking**	237 (60.9)	126 (56.8)	111 (66.5)	0.20
**Negative effects of medicines:**				
**inhalers causing addiction**	174 (47.7)	85 (38.2)	89 (53.3)	0.004
**inhaler side effect**	216 (55.5)	116 (52.2)	100 (59.9)	0.15
**inhaled steroids’ side effect**	295 (75.8)	176 (79.2)	119 (71.3)	0.07
**Treatment by herbs**	259 (66.5)	137 (61.7)	122 (73.1)	0.02
**Parents’ received education on asthma**	191 (49.1)	134 (60.3)	57 (34.0)	0.001

*χ^2^ test, *P* < 0.05 was considered significant.

**Table 3:** Asthma medications used by the surveyed Lebanese children

Medication	No. (%)
Antihistamines	90 (40.9)
β-agonists	81 (36.8)
Inhaled steroids	52 (23.6)
Oral steroids	39 (17.7)
Antitussives/mucolytics	38 (17.3)
Oral β-agonists	30 (13.6)
Leukotreine antagonists	29 (13.2)
Theophyllines	21 (9.5)
Long acting β-agonists	8 (3.6)

**Table 4:** Factors and parental perception that influence concerns toward the use of inhaled therapy in children with asthma (n = 343)

	No. (%) of parents who had concern with inhaled therapy		P*
Factor	yes (n = 216)	no (n = 127)	
Socioeconomic class:^†^			
class I	33 (15.6)	30 (24.0)	0.08
class II	64 (30.3)	43 (34.4)	0.47
class III	114 (54.0)	52 (41.6)	0.04
Settlement size:			
rural	100 (46.3)	41 (32.3)	0.01
urban	116 (53.7)	86 (67.7)	0.01
Preference for oral route	128 (77.1)	49 (49.5)	0.001
Worrying about inhaler causing addiction	143 (66.2)	26 (20.4)	0.001
Worrying about inhaled steroids use	206 (95.4)	80 (63.0)	0.001
Having received asthma education	104 (48.2)	80 (63.0)	0.0135

*χ^2^ test, *P* < 0.05 was considered significant.

†Class I – highly skilled workers (ie, physician, manager, teacher, pharmacist, shopkeeper); Class II – skilled workers (ie, clerk, factory foreman); Class III – semi-skilled and non-skilled worker (ie, taxi driver, factory workers, salesman, porter, laborer, waiter).

## Discussion

The majority of the surveyed parents did not recognize asthma by its name but called it chest allergy or recurrent dyspnea. The reason for this should be explored and any social stigma related to the disease discussed with the parents and their children. Moreover, most of the parents believed that asthma was a hereditary disease and some thought it was contagious.

This is the first study on parental asthma perception in a Lebanese population. Studies from the USA and India also showed that asthma was mostly perceived as a hereditary disease (17,18), while a study from Pakistan showed that it was mostly perceived as a contagious disease (1). The discrepancies between populations in the perception of asthma etiology are probably related to educational level and the quality of information provided by the treating physicians. In order to effectively treat children with asthma, it is important to explain to the caregiver that asthma is not solely hereditary but there are environmental factors that play a major role in asthma susceptibility and triggering of the attacks.

This study provides evidence that parents of children with asthma harbor considerable misperceptions about the disease. More than half of the surveyed parents expressed a lack of understanding or confusion about asthma etiology. Almost two thirds of parents reported that their children with asthma lived with someone who smokes, although tobacco smoke is identified as a significant trigger and exacerbating factor for asthma (19,20). In some other studies, parents of children with asthma have reported the use of complementary and alternative treatment such as massage, relaxation technique, diet (21), and the Echinacea herb (22). In our study, more than two thirds of parents thought that herbs had a role in the disease treatment. This is a worrisome finding given the lack of strong evidence on its benefits (21) and frequent side effect of herbal medications (22)

Parents of children with asthma often express a concern about the safety of asthma medications. Chan et al (11) reported that 66% of the parents were concerned about the side effects of asthma medications (91%), inhaler dependency (86%), cost of the inhaler (34%), and difficulty of its use (15%). Also parental worries about medication dependence were reported as a reason to wean off the children from medications (17). In our study, a half of the parents were worried about addictiveness of the inhaler and the majority of them worried about inhaled steroids’ side effects. Such prevalent worries may negatively impact asthma management and lead to serious sequels, which is why it is necessary that they are addressed by physicians. Moreover, parents who denied that their children had asthma and those from rural areas were more worried about inhaled therapy than those who accepted that their children had asthma and residents of urban areas. This will probably have an impact of non-adherence to treatment (11).

Our study found that about a half of the parents were from the low socioeconomic class, which is in accordance with the findings of a large community-based study (23). We also found that asthma in Lebanon was an important cause for school absence. Similar findings were shown in Chicago and Scotland (24,25).

This survey showed that Lebanon children more often used antihistamines than inhaled β-agonists and inhaled steroids for asthma control, as was shown in other studies (17). This is probably due to concerns about potential side-effects that led many parents to deviate from prescribed treatment regimens and use a number of over-the-counter medicines, such as antitussives and mucolytics, to control the symptoms. The parents who were concerned about the inhaled therapy significantly more often preferred oral route than the inhaler, even though they had a higher education. Such parental misconceptions should be addressed by adequate education, which is essential for better asthma control (26).

Parental education is also essential for ensuring a successful adherence to a treatment regimen. It is important to stress the role of medications in asthma control because parents often do not relate medication use to preventing asthma attacks. This should lead health care providers to investigate how parents’ beliefs may affect asthma control (9,26-28). Asthma patients and their guardians must understand and cooperate in the new approach to asthma care and control, and be willing to change behaviors according to the National Institute of Health guidelines (29).

There are several limitations to our study. The number of asthma cases may have been overestimated due to self-reported diagnosis and the public belief that recurrent dyspnea and chest allergy are equivalent to asthma. On the other hand, it might have been underestimated due to insufficient parental education and denying that their child had asthma. In addition, we were not able to validate the prescribed medication or correct the diagnosis by medical record review.

In conclusion, this survey revealed that parents of children with asthma harbored considerable misperceptions about the disease. Those who had most misperceptions on asthma etiology, triggers, and treatment modalities including inhaled therapy and corticosteroids were mainly parents from rural areas and parents who denied the label of asthma. Also, high rates of school absence and hospitalization due to asthma indicate poor asthma control in Lebanon. To improve asthma care and compliance among children it is necessary to provide adequate education to parents.
