# Supplementary material for: Parental perceptions and beliefs about childhood asthma: a cross-sectional study
Source: Croat Med J. 2011 Oct;52(5):637–43. doi: 10.3325/cmj.2011.52.637 (PMC3195973; doi:10.3325/cmj.2011.52.637)
Supplement: Supplementary material 1 [file CroatMedJ_52_s003.pdf]

Questionnaire about Parental Perceptional and Beliefs about Childhood Asthma

(Translated from the original Arabic version)

<table>
<tr><td colspan="6">Please check on the appropriate response</td></tr>
<tr><td>I. Parent's demographic filling the questionnaire :</td><td>Gender:
Male ( )
Female ( )</td><td>Age in years:
( )</td><td colspan="2">Residence:
Urban area ( )
Rural area ( )</td><td>Occupation: (Please specify)
………………..</td></tr>
<tr><td>Do you believe your child have any one of?</td><td colspan="2">Asthma ( )</td><td colspan="2">Chest Allergy ( )</td><td>Recurrent Dyspnea ( )</td></tr>
<tr><td colspan="6">If yes, please proceed to the next section</td></tr>
<tr><td colspan="3">II.Child's gender:
Male ( )
Female ( )</td><td colspan="3">Age in years:
( )</td></tr>
<tr><td colspan="6">Does your child have a regular (every 1-3 months) visit to the physician?
Yes( ) No ( )</td></tr>
<tr><td colspan="6">Have your child ever been hospitalized due to Asthma?
Yes( ) No ( )</td></tr>
<tr><td colspan="6">Have your child ever been absent from the school due to Asthma?
Yes ( ) No ( )</td></tr>
<tr><td colspan="6">Does your child have exercise related shortness of breath?
Yes ( ) No ( )</td></tr>
<tr><td colspan="6">Does your child have trouble breathing during sleep more than one night per week?
Yes ( ) No ( )</td></tr>
<tr><td colspan="6">Does your child have daily cough?
Yes ( ) No ( )</td></tr>
<tr><td colspan="6">Does your child have dyspnea at rest?
Yes ( ) No ( )</td></tr>
<tr><td colspan="6">Do you consult the pediatrician about your child's asthma attack?
Yes ( ) No ( )</td></tr>
<tr><td colspan="6">Are you able to treat your child's asthma attack at home?
Yes ( ) No ( )</td></tr>
</table>

What do you thing about the etiology of asthma?<br>Hereditary ( ) Contagious ( ) Others ( )
What do you believe about asthma trigger? :<br>Viral illness( ) Dust ( ) Food ( ) Indoor smoking ( )
Does your child receive any medication for Asthma, chest allergy, recurrent dyspnea?<br>Yes ( ) No ( )<br><br>**If yes pleas proceed to the next section**
What is/are your child's medication(s)? Please list<br>-<br>-<br>-<br>-
What is the preferred route for your child's medication?<br>Oral ( ) Inhaled ( ) Others ( )
Have you ever received education from physician about your child's medications?<br>Yes ( ) No ( )
Do you believe that inhaler may cause addition?<br>Yes ( ) No ( )
Are you worried about inhaler side effects?<br>Yes ( ) No ( )
Are you worried about inhaler steroid side effects?<br>Yes ( ) No ( )
Have you ever treat your child with herbs for asthma?<br>Yes ( ) No ( )
IV. Do you believe your child have an asthma diagnosis?<br>Yes ( ) No ( )
